# Supplementary material for: Description and Comparative Genomics of Algirhabdus cladophorae gen. nov., sp. nov., a Novel Aerobic Anoxygenic Phototrophic Bacterial Epibiont Associated with the Green Alga Cladophora stimpsonii
Source: Life (Basel). 2025 Feb 21;15(3):331. doi: 10.3390/life15030331 (PMC11943960; doi:10.3390/life15030331)
Supplement: Supplementary file 1 [file life-15-00331-s001.zip › life-3416383-supplementary.pdf]

Description and comparative genomics of *Algirhabdus cladophorae* gen. nov., sp. nov., a novel aerobic anoxygenic phototrophic bacterial epibiont associated with isolated from the green alga *Cladophora stimpsonii*.

Olga Nedashkovskaya [1*], Sergey Baldaev [1], Alexander Ivaschenko [2], Evgenia Bystritskaya [1], Natalia Zhukova [3], Andrey Kukhlevskiy [3], Valeria Kurilenko [1], and Marina Isaeva [1*]

[1] G.B. Elyakov Pacific Institute of Bioorganic Chemistry, Far Eastern Branch, Russian Academy of Sciences, Prospect 100 Let Vladivostoku 159, Vladivostok 690022, Russia; olganedashkovska@piboc.dvo.ru (O.N.); baldaevsergey@gmail.com (S.B.); ep.bystritskaya@yandex.ru (E.B.); valerievk141075@gmail.com (V.K.); issaeva@gmail.com (M.I.)
[2] Far Eastern Federal University, Suhanova Street 8, Vladivostok 690950, Russia; sashann2003@gmail.com (A.I.)
[3] A.V. Zhirmunsky National Scientific Center of Marine Biology, Far Eastern Branch, Russian Academy of Sciences, Palchevskogo Street 17, Vladivostok 690041, Russia; nzhukova35@list.ru (N.Z.); ad.kukhlevskiy@gmail.com (A.K.)
* Correspondence: olganedashkovska@piboc.dvo.ru (O.N.); issaeva@gmail.com (M.I.)

Supplementary

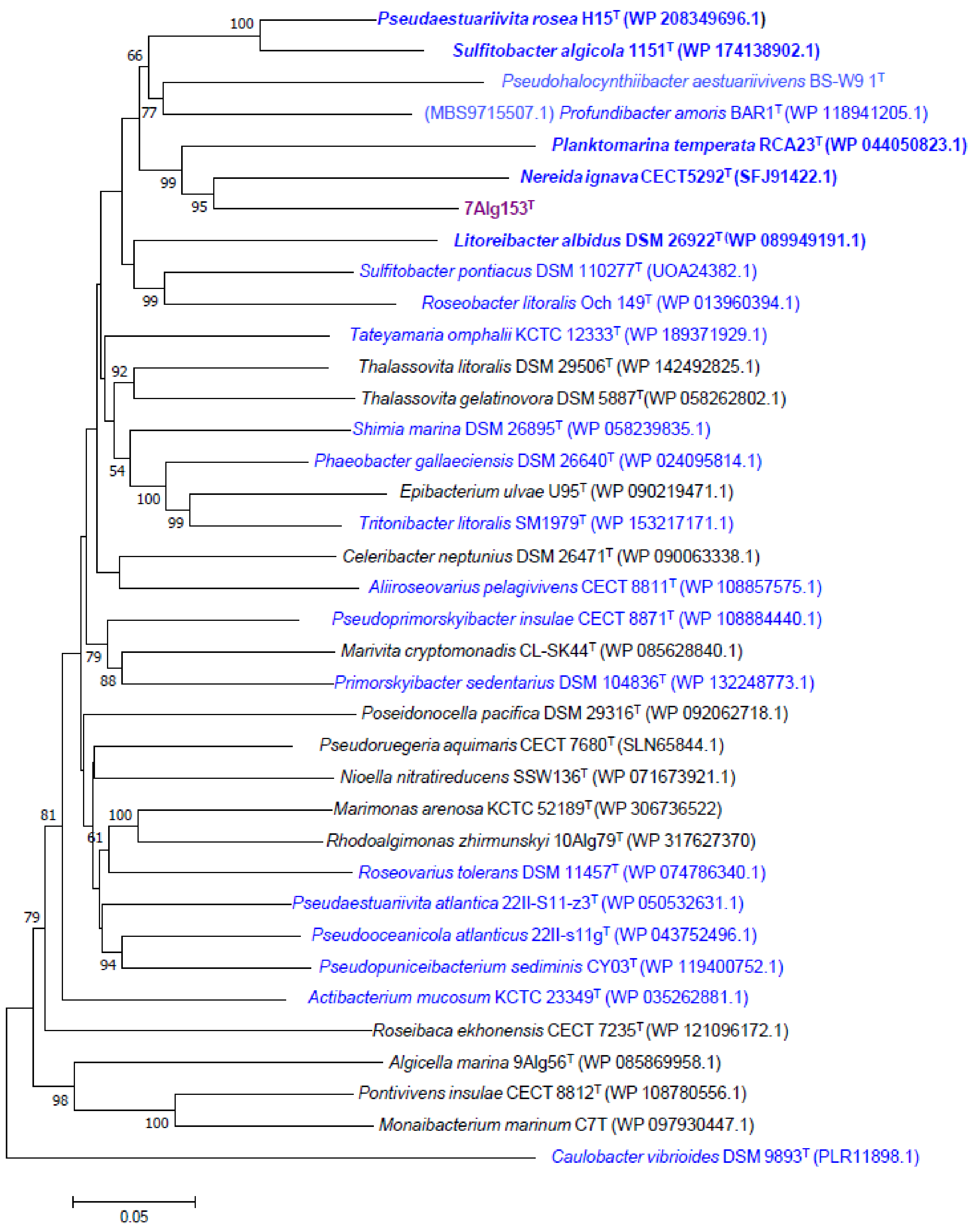

Figure S1. NJ RpoC tree showing phylogenetic relationships of the new strain 7Alg 153^{T} (KMM 6494^{T}) and type species strains from the closely related *Roseobacteraceae* and *Paracoccacea* (both formerly *Rhodobacteriaceae*) genera. Bootstrap values are based on 500 replicates and shown as percentage greater than 50. Bars are 0.05 substitutions per amino acid position. Strain *Caulobacter vibrioides* DSM 9893^{T} was used as an outgroup. GeBank/EMBL/DDB accession numbers are given in parentheses. The genomes of type species strains taken for a phylogenomic tree construction are shown in blue color.

(A)

	7Alg 153	*Litoreibacter albidus* DSM26922	*Nereida ignava* DSM 16309	*Sulfitobacter pontiacus* DSM 10014	*Pseudaestuariivita atlantica* 22II-S11-z...	*Planktomarina temperata* RCA23	*Sulfitobacter algicola* 1151T	*Pseudaestuariivita rosea* H15
7Alg 153	100	76	75	75	75	74	75	75
Litoreibacter albidus DSM 26922	76	100	76	75	75	73	74	75
Nereida ignava DSM 16309	75	76	100	75	74	74	73	74
Sulfitobacter pontiacu. DSM 10014^T	75	75	75	100	77	73	75	75
Pseudaestuariivita atlantica 22II−S11−z3^T	75	75	74	77	100	73	75	76
Planktomarina temperata RCA23^T	74	73	74	73	73	100	74	73
Sulfitobacter algicola 1151^T	75	74	73	75	75	74	100	85
Pseudaestuariivita rosea H15^T	75	75	74	75	76	73	85	100

(B)

	7Alg 153	*Nereida ignava* DSM 16309	*Litoreibacter albidus* DSM26922	*Pseudaestuariivita atlantica* 22II-S11-z3	*Sulfitobacter pontiacus* DSM 10014	*Planktomarina temperata* RCA23	*Sulfitobacter algicola* 1151T	*Pseudaestuariivita rosea* H15
7Alg 153^T	100	66	63	64	63	63	65	65
Nereida ignava DSM 16309^T	66	100	63	64	63	63	65	65
Litoreibacter albidus DSM 26922^T	63	63	100	62	63	61	63	62
Pseudaestuariivita atlantica 22II−S11−z3^T	64	64	62	100	64	62	62	63
Sulfitobacter pontiacu DSM 10014^T	63	63	63	64	100	61	62	62
Planktomarina temperata RCA23^T	63	63	61	62	61	100	61	61
Sulfitobacter algicola 1151^T	65	65	63	62	62	61	100	75
Pseudaestuariivita rosea H15^T	65	65	62	63	62	61	75	100

Table S1. The genome relatedness indexes display pairwise values of: (**A**) average nucleotide identities (ANI); (**B**) average amino acid identities (AAI) in percentages calculated using online servers ANI/AAI-Matrix, respectively.

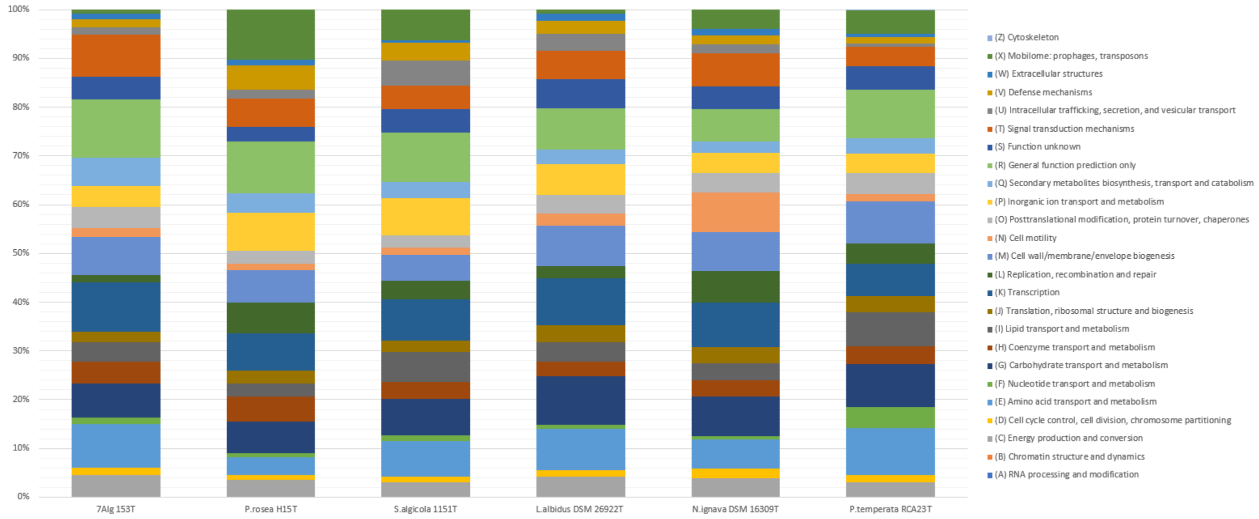

Figure S2. COG20 categories predicted in singletons among strains of the 7Alg 153^T clade.

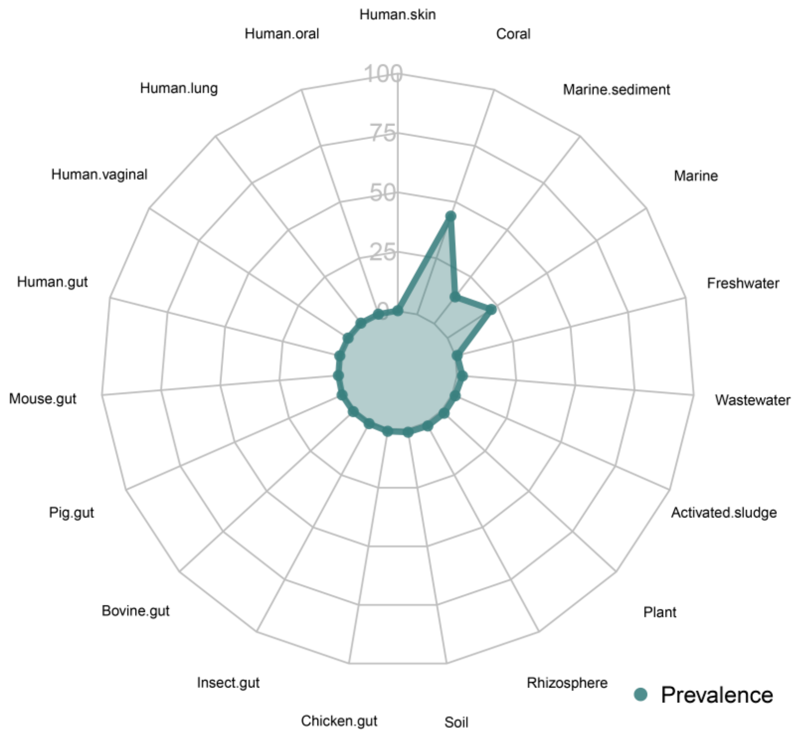

FigureS3. Distribution and habitat preferences of 7Alg 153^{T} based on 16S rRNA gene amplicon databases from Protologger.

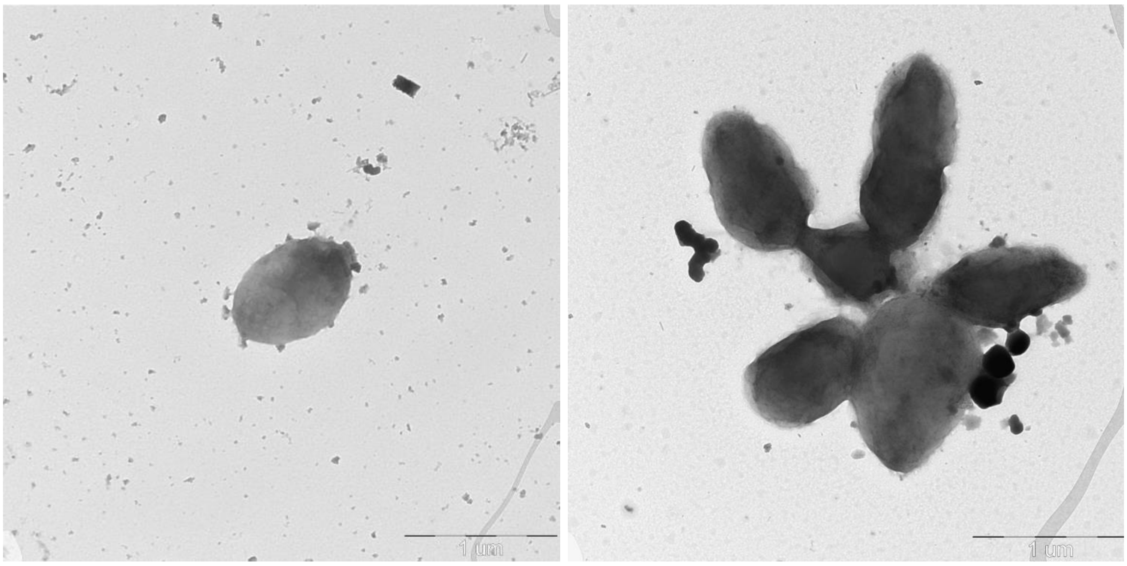

Figure S4. Transmission electron micrograph of strain 7Alg 153^{T}, bar, 1 μm.

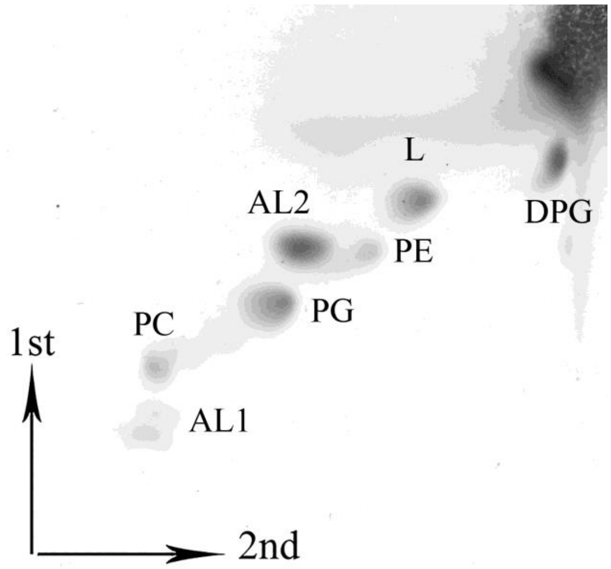

Figure S5. Two-dimensional thin-layer chromatograms of polar lipids of 7Alg 153^T. Abbreviations: DPG, diphosphatidylglycerol; PG, phosphatidylglycerol; PC, phosphatidylcholine; PE, phosphatidylethanolamine; AL1, AL2, unidentified aminolipids; L, unidentified lipid.
